# *C. elegans* as a tractable infection model for the emerging fungal pathogen *Candida auris*

**DOI:** 10.1128/spectrum.03156-25

**Published:** 2026-01-15

**Authors:** Melissa Martinez, Melissa R. Cruz, Danielle A. Garsin, Michael C. Lorenz

**Affiliations:** 1 The University of Texas MD Anderson Cancer Center UTHealth Houston Graduate School of Biomedical Sciences 4002, Houston, Texas, USA; 2 Department of Microbiology and Molecular Genetics, The University of Texas Health Science Center at Houston, McGovern Medical School 12339 , Houston, Texas, USA; University of Florida College of Dentistry, Gainesville, Florida, USA

**Keywords:** fungal pathogenesis, *Candida auris*, animal models, *C. elegans*

## Abstract

*Candida auris* is an emerging multidrug-resistant fungal pathogen. The genetic factors contributing to the virulence, drug resistance, and stress-tolerant nature of *C. auris* are mostly unknown. Additional animal models of virulence are needed, especially those amenable to high-throughput analysis. The nematode *Caenorhabditis elegans* has been validated as an effective tool for studying multiple fungal and bacterial pathogens. We describe here a *C. elegans* infection model in which exposure to *C. auris* is lethal to worms with kinetics similar to killing by *Candida albicans*; in contrast to *C. albicans*, *C. auris* does not form hyphae, indicating distinct virulence mechanisms. Furthermore, an engineered mutant auxotrophic for adenine biosynthesis (*ade2*∆) is avirulent, as it is in many models, indicating that the nematode can discriminate between virulent and avirulent mutants. Moreover, the *C. elegans* model can recapitulate strain-to-strain differences in virulence seen in mouse models. We have adapted a live/dead staining methodology using SYTOX Orange to enable a high-throughput assay amenable to analysis of multiple strains or genetic mutants. This model has significant advantages, including cost-effectiveness, a short generation time, and excellent amenability to high-throughput assays. *C. elegans* represents a valuable preliminary screening platform for *C. auris* virulence studies.

**IMPORTANCE:**

*Candida auris* is a growing clinical problem. This fungal pathogen spread rapidly worldwide after its discovery in 2009. It avidly colonizes the skin and abiotic surfaces, and many strains are multidrug resistant, making them easy to spread in hospital settings and very difficult to treat. Though distantly related to *Candida albicans*, it is clear that *C. auris* possesses unique virulence mechanisms, making studies directly in this species imperative. However, existing animal models, including mouse and invertebrate species, have limitations in variability and throughput. We describe here a facile infection model using the nematode *Caenorhabditis elegans*, which has previously been used for other bacterial and fungal pathogens. We show that this model can identify virulence differences between strains and in mutants predicted to be less virulent. Moreover, this model is amenable to high-throughput screening. This will be a valuable tool in uncovering *C. auris*-specific virulence traits.

## INTRODUCTION

*Candida* (*Candidozyma*) *auris* is an emergent fungal pathogen, first described in early 2009, that has rapidly risen in incidence as a highly transmissible hospital-acquired infection that primarily afflicts those who are immunocompromised (1–3). *C. auris* is particularly concerning due to its multidrug resistance, with about 90% of isolates resistant to some azole antifungals, 30% to amphotericin B, and 5%–10% to echinocandins (CDC). *C. auris* is particularly difficult to eradicate from healthcare facilities because it withstands standard disinfection protocols and persists on both abiotic surfaces and skin better than other fungal pathogens (4–7). The mortality rate for immunocompromised patients with an invasive *C. auris* infection can be as high as 60%, while having a chronic illness alongside the infection can increase mortality to rates as high as 80% (8–15). Initially identified in Japan, four distinct genomic clades rapidly arose that were initially geographically concentrated in East Asia, South Asia, Southern Africa, and South America (1, 16). There are now potentially six clades of *C. auris* (including isolates from Iran and Thailand) with several circulating in the United States and Western Europe, thanks to global travel (17–23). Because of its growing incidence and widespread drug resistance, the World Health Organization has classified *C. auris* as a critical fungal pathogen threat.

Most of what we understand about *C. auris* is inferred mainly from its more well-characterized but distant relative, *Candida albicans* (24–27). This distance is reflected in the recent reclassification of *C. auris* as *Candidozyma auris*, though *C. albicans* and *C. auris* are both part of the monophyletic CTG clade that includes several fungal pathogens. These fungi share conserved virulence factors, such as adhesins, proteases, and some genetic mutations that result in drug resistance (26, 28–32). However, significant differences distinguish *C. auris* from other *Candida* species. Most notably, while *C. albicans* is a dimorphic fungus, *C. auris* exists almost exclusively as a yeast, occasionally forming pseudohyphae under specific conditions (33–35). Indeed, the multidrug resistance common to *C. auris* is rare in other *Candida* species (26, 36–38). There is substantial phenotypic variation that arises from the genomic diversity both within and between clades (16, 37–41). This includes virulence in animal models, cellular aggregation, persistence on the skin, and drug resistance (16, 23, 42–45). The genetic factors that give rise to this diversity are largely unknown. Patient risk factors for developing *C. auris* infections overlap with other common nosocomial pathogens, but the persistence on the skin and abiotic surfaces and the resistance to some hospital disinfectant protocols facilitate person-to-person transmission that is rare among fungal pathogens (5, 46). We currently lack even a rudimentary understanding of the molecular basis of these enhanced challenges.

Studies using vertebrate and invertebrate animal models, including mice, *Galleria mellonella*, *Drosophila melanogaster*, and zebrafish, have shown that virulence and infectivity vary both between and within clades (41, 42, 47–51). Relative to well-established murine models for *C. albicans*, establishing a *C. auris* infection in mice requires either significant immunosuppressive regimes, a higher inoculum (100 times or more than that of *C. albicans*), or both (41, 52–55). Additional animal models, especially those amenable to high-throughput studies, are needed to bridge the knowledge gaps in *C. auris* virulence.

The nematode *C. elegans* has been established as a model for studying many human bacterial and fungal pathogens like *Pseudomonas aeruginosa*, *Salmonella enterica*, *Enterococcus faecalis*, *Cryptococcus neoformans*, and *Candida albicans*. Our group has used the nematode extensively to characterize aspects of *E. faecalis* pathogenesis and the antifungal properties of peptides derived from an *E. faecalis* bacteriocin, including initial findings with *C. auris* (56–59).

In this work, we sought to further characterize and expand the utility of *C. elegans* as an infection model for *C. auris*. We found that *C. auris* can establish a tractable infection in the worm with similar kinetics to that of *C. albicans*. This model can distinguish between a wild-type strain and engineered mutants predicted to be avirulent. It is also amenable to a medium-throughput 96-well plate-based assay using a live/dead stain and automated image analysis. The ease of inoculation via feeding, low maintenance cost, and high-throughput capability make the nematode an appealing model for studying *C. auris*, a genetically diverse and largely uncharacterized organism. Our findings suggest many opportunities for applying this model more widely as a screening tool to understand the unique virulence traits of this concerning pathogen.

## RESULTS

### *C. auris* infects and kills the nematode with kinetics similar to *C. albicans*

**Fig 1:**
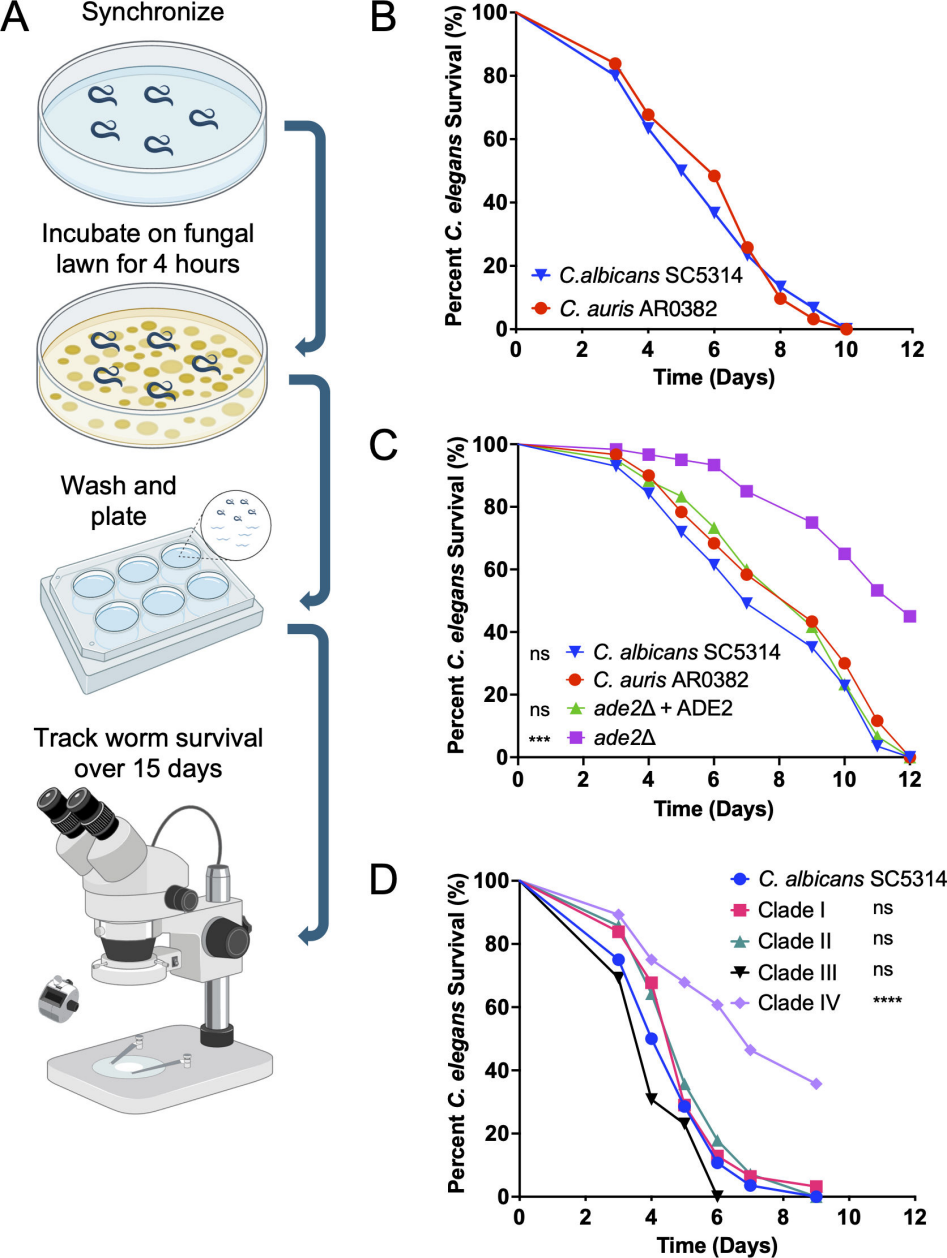
*C. auris* can establish an infection in the nematode with kinetics similar to *C. albicans*. (**A**) Schematic of the canonical nematode survival assay. Worms are synchronized to the L4 stage, then incubated on a fungal lawn for 4 hours at 25°C. After incubation, the worms are washed and transferred to liquid medium in a polystyrene plate, where survival is monitored daily. (**B**) *C. auris* AR0382 and *C. albicans* SC5314 kill *C. elegans* with similar kinetics, with all animals succumbing to infection within 10–12 days. (**C**) In the nematode model, the *C. auris ade2*∆ mutant demonstrates significantly reduced virulence compared to both the parent and complemented strains. The statistical comparisons indicated are to AR0382. ∗∗∗∗*P* < 0.0001; ns, not significant. (**D**) The nematode model can distinguish between *C. auris* isolates of varying virulence. The isolate from clade IV is significantly attenuated compared to the other strains tested.

We know from previous work that *C. albicans* can colonize the nematode intestine, subsequently undergoing hyphal morphogenesis and tissue invasion that ultimately results in the animal’s death (56, 59–61). In this study, we sought to determine whether *C. auris* could similarly establish an infection. We used *C. auris* clinical isolate AR0382 (clade I) from the Antimicrobial Resistance Bank at the Centers for Disease Control and Prevention. We chose this strain because it was previously used in several studies, is virulent in several animal models, and is amenable to genetic modification (62–64). For comparison, we used *C. albicans* SC5314, a well-characterized laboratory strain for which a *C. elegans* infection model was previously developed (60, 61). We used a similar infection protocol that involved feeding on lawns of *C. auris* for 4 hours (Fig. 1A)*,* followed by washing and transfer into liquid medium. *C. elegans* survival was then tracked daily, with dead worms being removed and recorded. The kinetics of nematode survival were similar to *C. albicans*; worms began to die by 4 days post-infection, and the median lethal time (LT_50_) of both strains was between 5 and 6 days (Fig. 1B).

A key feature of any infection model is its ability to distinguish between strains with different virulence profiles. To test whether *C. elegans* can differentiate between strains of *C. auris* with varying levels of virulence, we created an *ade2*∆ mutant in the AR0832 background using CRISPR/Cas9. The *ADE2* gene encodes the enzyme phosphoribosylaminoimadazole carboxylase, a key enzyme of adenine biosynthesis; purine metabolism is required for virulence in essentially all pathogens in which it has been tested, including both *ade2*∆ and *ura3*∆ mutants in *C. albicans* and *C. neoformans* (65–68). As expected, the *ade2*Δ strain does not grow in media lacking adenosine or adenine (not shown). It shows a modest growth defect compared to the parent strain (Fig. S1A) when grown in nutrient-poor worm medium (80% M9/20% brain-heart infusion [BHI]; see Materials and Methods). There is no growth defect in nutrient-replete yeast extract-peptone-dextrose (YPD) medium (Fig. S1B). We found that the LT_50_ of the mutant was 10 days, compared to approximately 6 days for the parent strain (Fig. 1C). This is a significantly greater attenuation in the worm model than seen for the highly avirulent *C. albicans cph1*∆ *efg1*∆ mutant (60, 61). The avirulence of the *ade2*∆ mutant in *C. elegans* confirms that this model can elucidate virulence differences between genetic variants in otherwise isogenic strains.

We further wanted to explore whether *C. elegans* can distinguish between different isolates of *C. auris*. We tested one isolate from each of the four major clades. In this assay, the isolate from clade IV was less virulent than isolates from the other three clades, which behaved similarly to the SC5314 strain. The data suggest that *C. elegans* can distinguish different levels of virulence between *C. auris* strains (Fig. 1D). This may represent inherent differences between clades in this model but is perhaps more likely to recapitulate strain-to-strain variation in virulence seen in other models, including in mice (39, 40, 49, 51, 52, 63, 64, 69).

### *C. auris* colonizes the intestine of *C. elegans* but does not form hyphae

**Fig 2:**
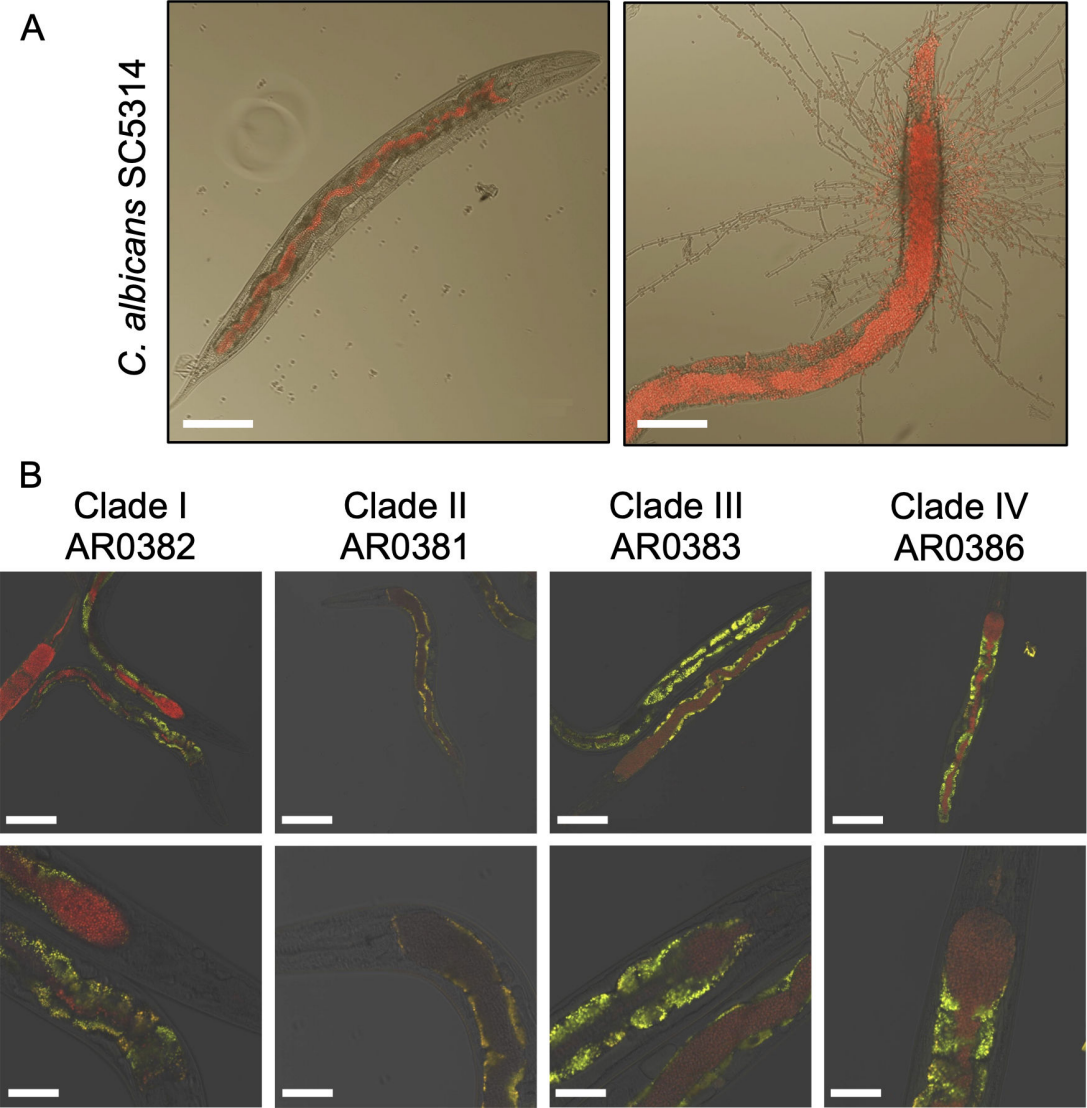
*C. auris* colonizes the worm gut without penetrating intestinal tissue. (**A**) *C. albicans* first colonizes the intestine, causing distention, followed by hyphal morphogenesis, which results in tissue invasion. Scale bars are 100 μm. (**B**) Nematodes infected with clinical isolates from the four major *C. auris* clades. The bottom row shows magnified views of the distended intestine. *Candida* cells are red from a fluorescent marker, and the intestinal cells are yellow/green due to autofluorescence. Scale bars: 120 μm (top row) and 40 micrometers (bottom row).

In previous studies, *C. albicans* was observed to establish an infection in *C. elegans* by colonizing the lumen of the intestine. Later in the infection, typically around 5–6 days post-inoculation, *C. albicans* begins to form hyphae, which penetrate the worm from the inside out (56, 60, 61, 70) and result in worm death, as the hyphae and/or pseudohyphae damage the intestinal wall and allow the fungus to invade surrounding tissues (Fig. 2A) (56, 60, 61). Like *C. albicans*, *C. auris* colonized the lumen of the worm intestine, distending the normal gut architecture (Fig. 2B). Though there is some evidence that *C. auris* can form pseudohyphae under certain conditions, we did not observe this occurring in the *C. elegans* intestine. Therefore, *C. auris* virulence in the worm appears to use mechanisms unrelated to tissue invasion, indicating significant differences relative to *C. albicans*. What these might be remains unknown, but *C. auris* has, for instance, a distinct set of novel adhesins like Scf1, which may contribute to colonization in this model (64). There were no qualitative differences in gut colonization across strains from each of the four major clades of *C. auris*.

### A *C. elegans* high-throughput assay to study *C. auris* infection  

**Fig 3:**
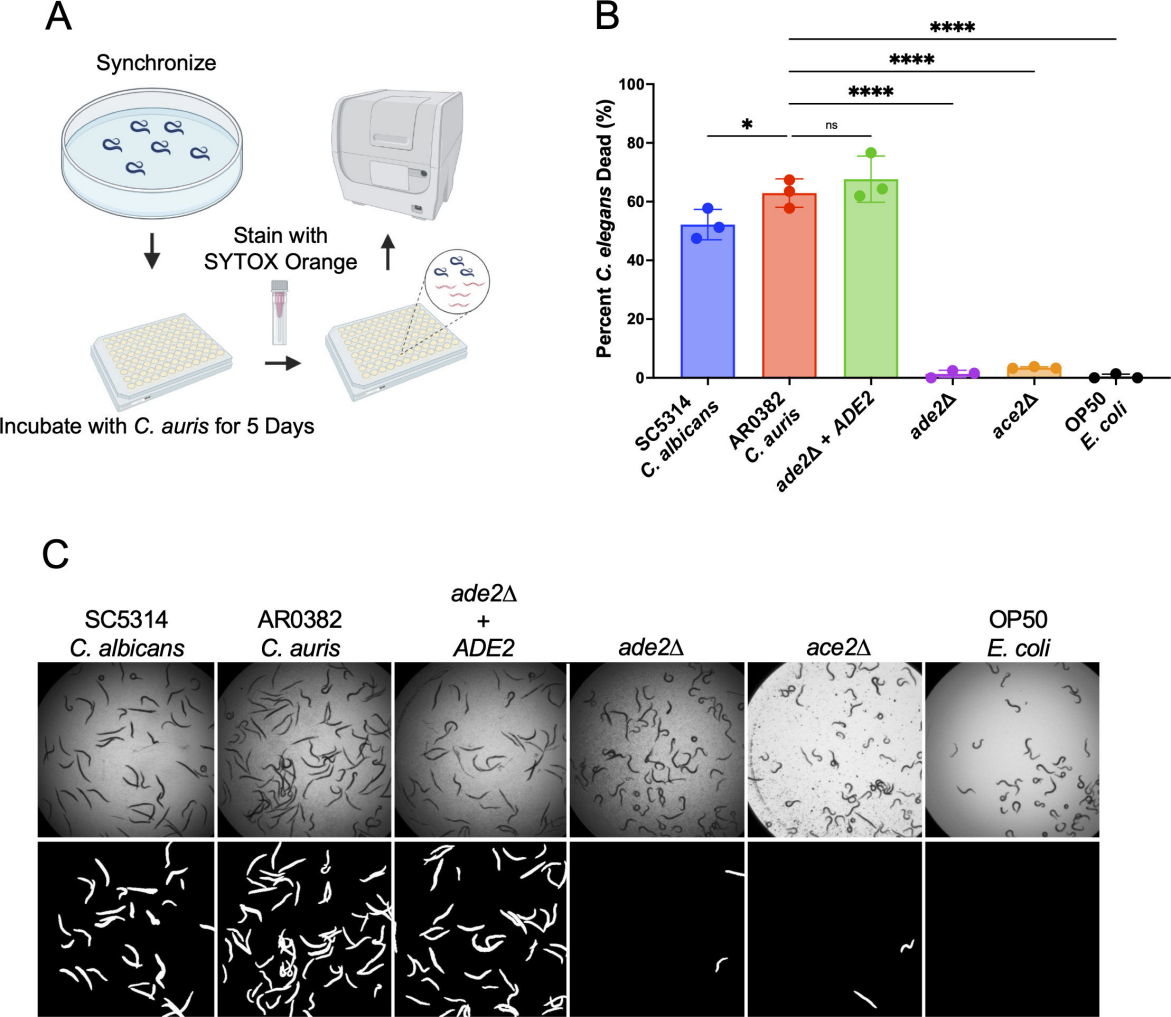
Results from canonical *C. elegans* survival experiments are recapitulated using a high-throughput assay. (**A**) Schematic of the high-throughput SYTOX Orange assay. Worms are synchronized, incubated with *C. auris* culture for 5 days, washed, stained with SYTOX Orange, and imaged using a plate reader. (**B**) The *C. auris ade2*∆ mutant shows reduced virulence compared to both the parent and complemented strains. ∗*P* < 0.05; ∗∗∗∗*P* < 0.0001; ns, not significant. (**C**) Representative images from the high-throughput SYTOX Orange assay. The top row shows pictures taken in bright field, and the bottom row contains fluorescent images processed by the CellProfiler analysis pipeline, as described (72), to normalize for image intensity and background.

Recent studies have utilized the nematode model in high-throughput assays, primarily focused on discovering novel antimicrobial agents (71–77). We wanted to determine whether the *C. elegans* model could be adapted to a high-throughput approach to investigate *C. auris* virulence. Rather than inoculation by feeding on a fungal lawn, a fungal inoculum is added to *C. elegans* in liquid medium, followed by a 5-day coincubation (Fig. 3A). The worms are then washed and stained overnight with SYTOX Orange, a nucleic acid-binding dye that stains dead worms for an easily visualized live/dead assay. The dead-to-total worms ratio is calculated using an automatic image analysis pipeline adapted from Conery et al. and used as a proxy for virulence (72). In this context, we sought to assess the virulence of the *ade2*Δ mutant and an *ace2*Δ mutant created by the O'Meara lab that had attenuated virulence in the *G. mellonella* model (64, 78). We found that the high-throughput assay recapitulated the results from the standard *C. elegans* survival assay, with the *ade2*Δ and *ace2*Δ mutants associated with significantly reduced worm death relative to the parent AR0382 strain (Fig. 1C and 3B). Qualitative data could also be derived from the images used in the pipeline. Representative bright-field and fluorescent images, processed via the CellProfiler pipeline (72), are shown in Fig. 3C. The virulence patterns of these mutants were similar to those observed with the OP50 *Escherichia coli* strain widely used to propagate *C. elegans* in the lab (Fig. 3C).

### Virulence phenotypes differentiating *C. auris* strains are recapitulated in the *C. elegans* model

**Fig 4:**
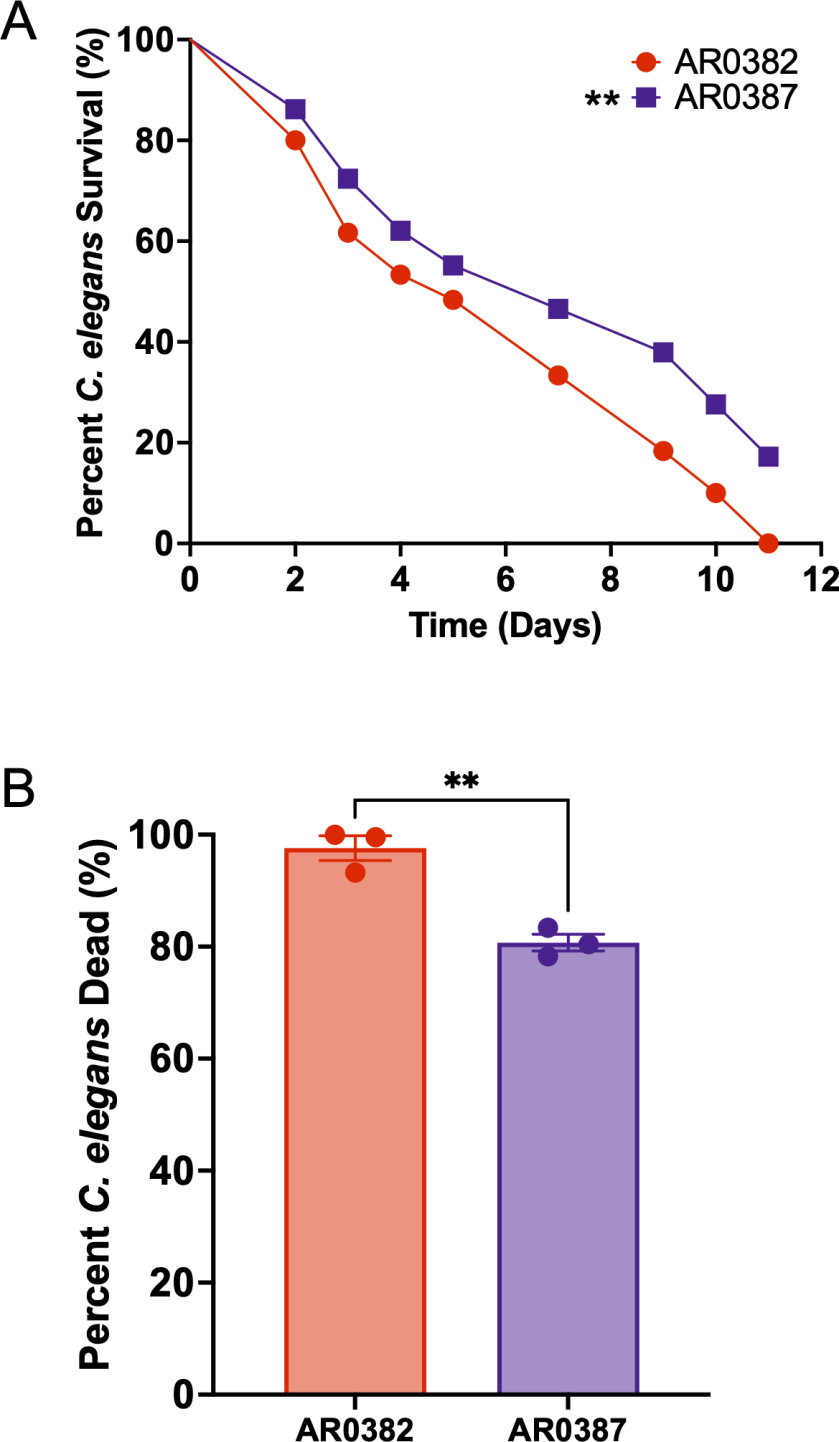
AR0387 is less virulent than AR0382 in the *C. elegans* model. (**A**) In the standard assay, *C. elegans* survival over time following exposure to *C. auris* AR0387 compared to AR0382. ∗∗*P* < 0.01. (**B**) Percent *C. elegans* dead following 5 days of exposure to AR0387 and AR0382 and assayed using the SYTOX Orange assay. ∗∗*P* < 0.01.

We aimed to assess whether our model could replicate the phenotypes observed in mammalian models, particularly in mice. Previously published studies using mice showed that AR0382 is more virulent than AR0387, another clade I isolate. Santana et al. report that mice infected with AR0382 succumbed within 15 days post-inoculation, while 50% of those infected with AR0387 survived beyond 20 days (64). Additionally, the fungal burden observed in mice differed between the two isolates; Vila et al. reported higher CFU counts in the kidneys of mice inoculated with AR0382 (48). When tested in *C. elegans* as shown in Fig. 4, worms infected with AR0382 succumbed more rapidly to infection, with an LT_50_ of 5 days, compared to 7 days for those infected with AR0387 (Fig. 4A). This difference in comparative virulence was also reflected in the SYTOX assay, where AR0382 was associated with significantly greater nematode death (Fig. 4B). These findings demonstrate that AR0382 is more infectious than AR0387 in the *C. elegans* model, which agrees with the mouse model (18, 30, 48, 64). These data further support *C. elegans* as a relevant model for *C. auris* infection and one with substantial experimental advantages.

## DISCUSSION

*C. auris* is a greatly concerning clinical problem that presents many mysteries. Its near-simultaneous emergence on multiple continents in at least four (and perhaps six) genetically distinct clades defies an easy explanation. While hypotheses have been mooted for its environmental reservoirs, drug resistance, and sudden emergence, none of these are backed by compelling evidence (79–82). Nevertheless, clinical cases are increasing exponentially in the United States and across the world (22, 23, 83–85). Containment and treatment of this emerging pathogen requires better experimental models to understand the disease potential of different strains and clades and the underlying genetic features that define them. We show here that *C. elegans* can be used as *a C. auris* infection model. One earlier study using *C. elegans* evaluated virulence differences among clinical isolates exclusively with a 5-day survival assay (47). Here, we expand on this approach by employing both an extended survival assay and a shortened, high-throughput-compatible assay. By tracking survival over time, we demonstrated that *C. auris* can establish an infection in the nematode with kinetics similar to those of *C. albicans*. In both species, this begins with gut colonization, but *C. auris* infection proceeds without the tissue penetration observed with *C. albicans*. A SYTOX-based live/dead fluorescence assay successfully recapitulated these results, providing the basis of high-throughput screening.

Importantly, a mutant in *ADE2*, known to strongly affect virulence in multiple microbial pathogens, was attenuated compared to the parent strain in both the survival and SYTOX assays. Deleting the *ADE2* gene in other pathogenic fungi like *C. albicans* and *C. neoformans* species was associated with dramatically reduced virulence (67, 68). The *C. auris ade2*∆ null mutant in our model was significantly less virulent than the parent strain, as expected. The *C. elegans* model also recapitulated strain variation in phenotypes observed in murine studies, with clinical isolate AR0382 being considerably more virulent than AR0387.

We suggest that *C. elegans* has an important role as a screening model for *in vivo* study of new and rapidly evolving pathogens like *C. auris*, complementing more complex and costly vertebrate models. Its advantages include the ability to propagate large numbers of isogenic animals quickly and cheaply in the laboratory environment, thanks to its short generation time (3 days), well-established genetics, and its successful use with many bacterial and fungal pathogens (86). The model can be used in a standard plate-based nematode survival assay, but we also showed that it is amenable to a high-throughput methodology using a SYTOX-based live/dead assay to test *C. auris* virulence. Furthermore, *C. elegans* provides a platform for *in vivo* imaging of microbial infections, offering valuable insights into the host-pathogen interaction, highlighted by the fact that *C. auris* does not invade the worm tissue, unlike *C. albicans*. This reinforces that the virulence mechanisms of these two distant relatives are likely to be quite different, and that *C. albicans* paradigms cannot be extrapolated to *C. auris* without direct study.

The *C. elegans* infection model is intended to augment rather than replace mammalian models. The nematode lacks an adaptive immune system and specialized immune cells, such as macrophages and neutrophils, which are essential components of the immune response during *Candida* infections in mammals. The *C. elegans* experiments are performed at a temperature below that of the mammalian body. However, we have shown that the nematode can effectively identify significant phenotypes, which can then be further investigated using mammalian models.

*C. elegans* offers a rapid and effective approach for determining potential virulence factors. It has been used to screen insertion mutant libraries in *P. aeruginosa*, *E. faecalis*, *C. neoformans*, and *C. albicans*, leading to the identification of both established and novel virulence factors (87–90). Beyond these pathogens, *C. elegans* has also been employed to study rare pathogens. For example, the novel virulence factor AidA was identified partly through *C. elegans* experiments in the *Burkholderia cenocepacia* cystic fibrosis isolate H111 (91). *Yersinia pestis* kills *C. elegans* through a biofilm-dependent mechanism, highlighting the versatility of this model in uncovering diverse pathogenic strategies (76). Our ultimate goal is to utilize the nematode to identify novel virulence factors in fungal pathogens. While the SYTOX assay has most commonly been used to identify and optimize novel antimicrobials in *C. albicans*, here we demonstrated that it can be used to test for virulence in *C. auris*, and this may have advantages in the genetic dissection of virulence in this haploid species.

## MATERIALS AND METHODS

### Microbial strains and media

For *C. elegans* maintenance and propagation, animals were fed *E. coli* strain OP50 on nematode growth medium agar using standard techniques (92). Fungal strains were routinely grown in YPD medium. *C. albicans* wild-type strain SC5314 was used as a control for most experiments (93). Mutants were generated in the AR0382 strain (a.k.a. B11109, CDC Antimicrobial Resistance Bank). *C. auris* was transformed using a modified CRISPR-Cas9 approach (63, 94–96). In short, CAS9 was PCR-amplified from pRB732. We selected specific guide RNAs and PCR-amplified them using pTO136 as a template. A repair construct was designed by flanking the nourseothricin resistance marker with approximately 500 base pairs targeting the gene *ADE2* (97). To create the complement strain, the *ADE2* gene was reintegrated via homologous recombination at the native locus. *C. auris* strains AR0381 (B11220, clade II), AR0382 (B11109, clade I), AR0383 (B11221, clade III), and AR0386 (B11245 IV) used for fluorescence microscopy were also obtained from the CDC Antimicrobial Resistance Bank. These strains were tagged with the fluorophore mScarlet as previously described (98). As previously described, *C. albicans* SC5314 was tagged with fluorophore dTomato (99).

### *C. elegans* survival assay

*C. elegans glp-4(bn2);sek-1(km-4)* were used for all experiments. The survival assays were performed as in early studies (56, 57, 61, 100). Infection plates were prepared by growing fungal strains in YPD liquid media for 24 hours at 30°C with shaking. Five hundred microliters of the culture was plated onto BHI solid media containing gentamycin at 10 μg/mL for 24 hours at 30°C. Synchronized L4 stage nematodes were decanted on the fungal lawns and allowed to graze for 4 hours. The nematodes were washed with M9 pipetted into six-well plates (approximately 30 worms per well, two wells per condition) containing 2 mL of 20% BHI 80% M9. Plates were incubated at 25°C, and worm death was scored daily.

### Fluorescence microscopy of infected animals

To investigate colonization of the intestine during infection, worms were infected with mScarlet and dTomato tagged strains as described above for the survival assay. After 6 days, live worms were washed with M9 and mounted on 2% agarose pads. The animals were imaged using an Olympus FLUOVIEW FV 300 confocal microscope imaging system and Fluoview FC315-SW software.

### High-throughput SYTOX Orange assay

The SYTOX Orange assay was performed as in earlier studies (58, 71–73). L4 stage nematodes were pipetted into a 96-well plate (about 30–50 worms per well, 3+ wells per condition in assays with controls only, one well per condition in isolate library screen) in 20% BHI 80% M9 plus gentamycin at 10 μg/mL concentration. In most assays, the wells were inoculated with fungal culture grown for 16 hours at 30°C with shaking and standardized to a final optical density at 600 nm of 0.03. For screening the isolate library, the strains were grown in 200 μL of YPD liquid media for 16 hours at 30°C with shaking in a 96-well plate. One microliter per condition was used to inoculate the worm plate. After 5 days, the plates were washed with M9 and stained with Invitrogen SYTOX Orange Nucleic Acid Stain (diluted 2 μL to 10 mL of M9). Plates were washed and imaged using a BioTek Cytation5 Imaging Reader. Fluorescence and bright-field images were taken of each well. The TRITC filter cube was used for fluorescent imaging. The automated Cell Profiler pipeline was used to determine the percentage of dead worms. This pipeline counts the black pixels in the bright-field image and red pixels in the fluorescent image. The ratio of red to black pixels determines the percentage of dead worms.

### Statistics

Kaplan-Meier survival curves were generated and compared using Mantel-Cox log-rank analysis. For SYTOX assays, unpaired *t*-tests or one-way ANOVA with multiple comparisons and Dunnett’s test were used to compare conditions. GraphPad Prism was used for statistical analysis. For all statistical tests, *P* values of <0.05 were considered statistically significant, and asterisks in the figure panels indicate the levels of significance as follows: *P < 0.05, **P < 0.01, ***P < 0.001, ****P < 0.0001.

## SUPPLEMENTAL MATERIAL

Data Set S1 spectrum.03156-25-s0001.xlsx Raw data for the figures.

Figure S1 spectrum.03156-25-s0002.pdf The *ade2*∆ mutant has a growth defect in worm media.

ASM does not own the copyrights to Supplemental Material that may be linked to, or accessed through, an article. The authors have granted ASM a non-exclusive, world-wide license to publish the Supplemental Material files. Please contact the corresponding author directly for reuse.
