# Supplementary material for: C. elegans as a tractable infection model for the emerging fungal pathogen Candida auris
Source: Microbiol Spectr. 2026 Jan 15;14(3):e03156-25. doi: 10.1128/spectrum.03156-25 (PMC12955389; doi:10.1128/spectrum.03156-25)
Supplement: Figure S1 — The ade2∆ mutant has a growth defect in worm media. [file spectrum.03156-25-s0002.pdf]

A

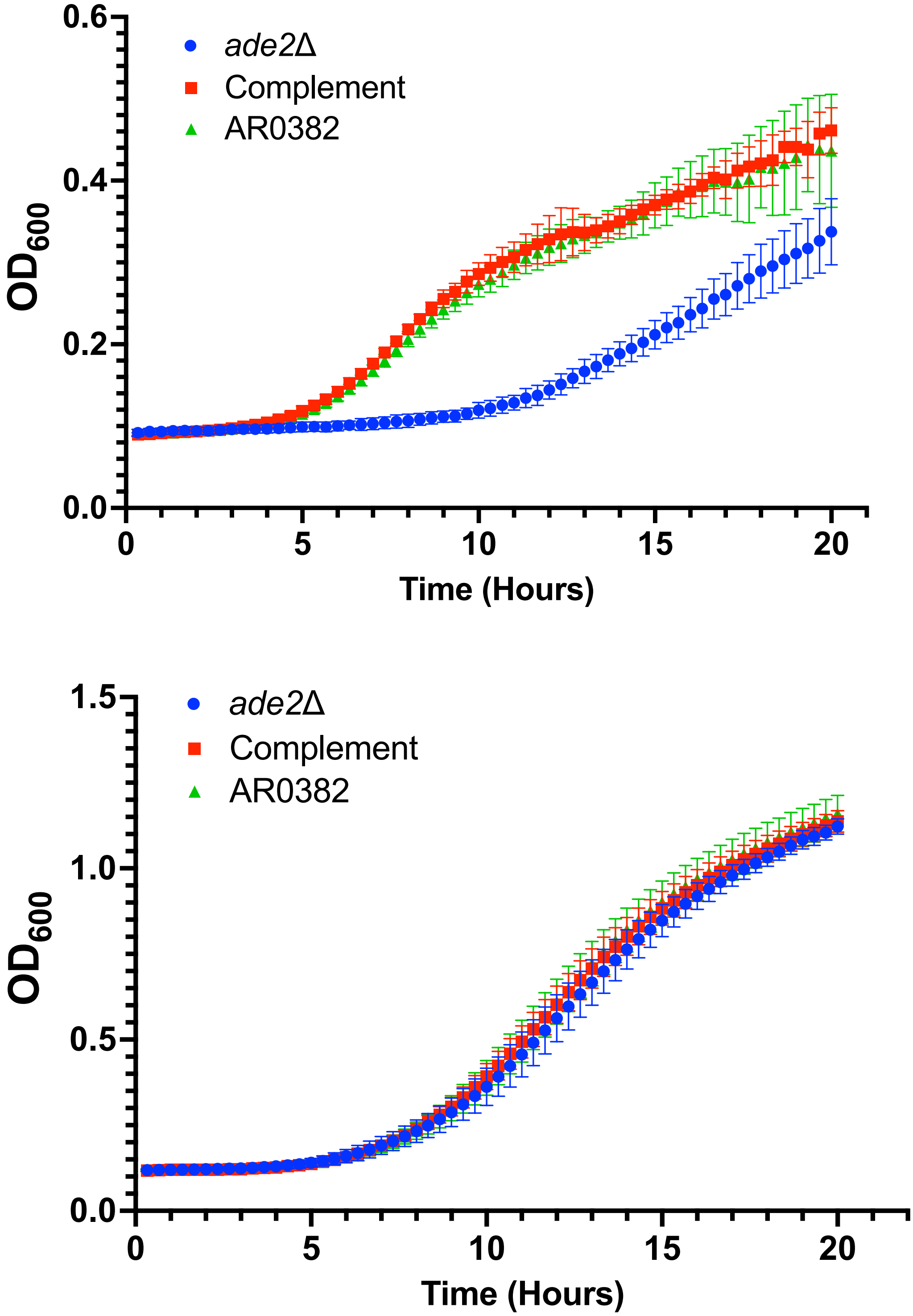

B

Supplemental Figure 1: The *ade2Δ* mutant has a growth defect in worm media.

A) 22-hour growth curve in 20% BHI 80%M9. The *ade2Δ* mutant grows at a slower rate than the complement and parent strain. **B)** 22-hour growth curve in YPD. The mutant, complement, and parent strains grow at a similar rate.
